# The Alliance of Mesenchymal Stem Cells, Bone, and Diabetes

**DOI:** 10.1155/2014/690783

**Published:** 2014-07-16

**Authors:** Nicola Napoli, Rocky Strollo, Angela Paladini, Silvia I. Briganti, Paolo Pozzilli, Sol Epstein

**Affiliations:** ^1^Division of Endocrinology and Diabetes, Università Campus Bio-Medico di Roma, Via Alvaro del Portillo 21, 00128 Rome, Italy; ^2^Division of Bone and Mineral Diseases, Washington University in St Louis, St Louis, MO, USA; ^3^Centre for Diabetes, The Blizard Building, Barts and The London School of Medicine, Queen Mary, University of London, London, UK; ^4^Division of Endocrinology, Mount Sinai School of Medicine, New York, USA

## Abstract

Bone fragility has emerged as a new complication of diabetes. Several mechanisms in diabetes may influence bone homeostasis by impairing the action between osteoblasts, osteoclasts, and osteocytes and/or changing the structural properties of the bone tissue. Some of these mechanisms can potentially alter the fate of mesenchymal stem cells, the initial precursor of the osteoblast. In this review, we describe the main factors that impair bone health in diabetic patients and their clinical impact.

## 1. Introduction

Bone fragility has emerged as a new complication of Type 2 Diabetes (T2D). The pathophysiological link between bone fragility and diabetes is not completely understood. Several mechanisms may influence bone homeostasis by impairing the function of osteoblasts, osteoclasts, and osteocytes and/or changing the structural properties of the bone tissue. Notably, adipocytes and osteoblasts are derived from a common precursor, the mesenchymal stem cell (MSC), and the differentiation is modulated by several interacting pathways that may be disrupted in diabetes. Other organs and endocrine systems such as the gut, kidney, and cardiovascular and vitamin D systems are altered in diabetes and, therefore, may also affect bone metabolism. As a result, fractures are an added burden in diabetes. However, while bone mineral density (BMD) is decreased in patients with Type 1 diabetes (T1D), it is normal or even increased in T2D patients.

In this review, we describe the main factors that impair bone health in diabetic patients and their clinical impact.

## 2. The Mesenchymal Stem Cell Fate: To Be or Not to Be

Although fat cells primarily compose the adipose tissue, they also populate bone marrow in coexistence with osteoblasts and their common mesenchymal progenitor [1]. A fine balance exists between adipogenesis and osteoblastogenesis that rely mainly on the activity and interdependence of two systems, the WNT signaling and the Peroxisome proliferator-activated receptors-*γ* (PPAR-*γ*) pathways [2]. Activation of Wnt signaling pathway promotes osteogenesis [3–5], while inhibiting adipogenesis [6, 7]; on the other hand, PPAR-*γ* favors the differentiation of mesenchymal stem cells into adipocytes over osteoblasts [8]. The reciprocal activity of these pathways may determine the prevalence of one lineage over the other, leading, for example, to impaired bone formation in case of prevailing adipogenesis. In fact, marrow adipogenesis have been associated with reduced, bone formation [9–11], and BMD [12, 13], the latter being a strong predictor of fracture risk [14].

### 2.1. An Osteoblast: The WNT Signaling Pathway

Wnt glycoproteins are a large family of growth factors (19 secreted proteins) that mediate crucial biological processes like embryogenesis, organogenesis, and tumorigenesis. The WNT signaling consists of the canonical (or Wnt/*β*-catenin) and the noncanonical pathways (reviewed in [15]). The Wnt/*β*-catenin pathway is activated when canonical Wnt ligands signal through the Frizzled receptors and the low-density lipoprotein receptor-related protein- (LRP-) 5 or LRP-6 coreceptors. The signal leads to inhibition of the glycogen synthase kinase (GSK) 3*β*. This serine/threonine kinase is a main regulator of *β*-catenin degradation and therefore activity. WNT activity is tightly regulated by several molecules acting locally. Those that interact with Wnt coreceptors LRP (e.g., sclerostin and Dickkoff or Dkk-1) [16–18] selectively inhibit the Wnt canonical pathway; others bind to Wnts, thus inhibiting both canonical and noncanonical signalling.

WNT canonical pathway controls MSC differentiation to three specific lineages, adipocytes, osteoblasts, and chondrocytes. For example, WNT canonical pathway represses chondrocyte [19] and adipocytes differentiation [20], but it is required for chondrocyte hypertrophy [19]. Moreover, the activation of the Wnt/*β*-catenin (primarily Wnt10b) promotes osteoblast differentiation and proliferation from MSC, through stimulation of osteogenic transcription factors, such as Runx2 and osterix [21]. This process activates a negative feedback control with Dkk-1 and sclerostin production by osteocytes. At the same time osteoblasts produce osteoprotegerin (OPG) which decreases osteoclast differentiation and maintains bone homeostasis [22]. Inhibition of adipogenesis by Wnt signaling pathway appears to be mainly mediated by *β*-catenin which inhibits expression of selected PPAR*γ*2 targets genes [23].

The relevance of WNT is also proved by the fact that mutations in the LRP-5 Wnt coreceptor are associated with changes in BMD [3, 4, 24]. For example, loss-of-function LRP-5 knock-out mice present reduced bone mass [3, 25], while point mutations in the LRP-5 gene are related to increase bone mass in humans [4] and mice [26, 27]. On the other hand, variants at the LRP-5 gene are strongly associated with obesity and LRP-5 knock-out mice showed increased plasma cholesterol and impaired glucose tolerance [28].

### 2.2. An Adipocyte: The PPAR-*γ*2 Pathway

Adipogenesis is under the control of the master regulator PPAR-*γ*. PPARs are transcriptional regulators that form part of the ligand activated nuclear hormone receptor superfamily [29]. PPAR-*γ* is expressed as two protein isoforms produced from a single gene [30, 31]. While the expression of PPAR-*γ*1 takes place in many different cell-types, PPAR-*γ*2 is expressed only in adipocytes and bone marrow stromal cell [32, 33].

PPAR-*γ*2 acts as a molecular switch that regulates the fate of pluripotent MSC. PPAR-*γ*2 upregulation during the early phases of adipogenesis directly relates with the presence of CCAAT/enhancer binding protein (C/EBP) family of transcription factors, which are stimulated by adipogenic hormones [29]. Growth factors signaling through the MAP kinase (MAPK) cascade may regulate PPAR-*γ*2 activity [34]. On the other hand, osteogenic stimuli that enhance Wnt signaling may inhibit PPAR-*γ*2 by formation of a corepressor complex [35]; reciprocally, PPAR-*γ*2 suppresses Wnt signaling by stimulating the degradation of *β*-catenin by proteasome [36].

This pathway is relevant and also being target of some antidiabetic drugs like Thiazolidinediones (TZDs) which are effective in lowering blood glucose but also increase the risk of fractures [37]. In fact, activation of PPAR-*γ*2 in bone marrow enhances MSC differentiation to adipocytes while inhibiting osteogenesis. Some studies have demonstrated an accumulation of lipids in bone marrow [38] and an increase of PPAR-*γ*2 expression associated with bone loss and aging [39]. Adipocyte-specific transcription factors such as PPAR*γ*2 and C/EBPa are more represented in old bone marrow than in adult marrow [39]. Similarly, mice with T1D display increased PPAR-*γ*2 in bone tissue, reduced bone formation, and increased marrow adiposity [40]. Abnormal expression of these factors increased accumulation of lipids in sites outside of adipose tissues such as the bone marrow by inducing differentiation of mesenchymal cells toward a mesenchymal adipocyte-like default cell (MAD cell) [39].

## 3. Osteoclasts and the RANKL System

Marrow stromal cells and osteoblasts are required for the formation and activation of osteoclasts. Osteoclast precursors come from the haematopoietic stem cell compartment and need to directly interact with molecules produced by osteoblasts such as M-CFS and the receptor activator of nuclear factor-*κ*B ligand (RANKL) [41]. RANKL is a member of the tumor necrosis factor (TNF) family which, upon binding to RANK, activates downstream signaling molecules in osteoclasts such as nuclear factor-*κ*B (NF-*κ*B), c-fos, MAPK, and TNFRAF6 that are involved in differentiation and activation [42]. RANKL signal induces also the activation of small GTP-ases that modulate cytoskeletal activity and thus function and survival of osteoclasts. Osteoclastogenesis is also downregulated by OPG, another osteoblast derived protein which works as decoy receptor for RANKL and prevents the RANK/RANKL interaction [43]. Glass et al. showed that OPG expression by osteoblasts was dependent on the activity of *β*-catenin, the main WNT signaling effector [44], while Wnt3a inhibits osteoclastogenesis at later stages [45]. Conversely, activation of noncanonical pathway by Wnt5a stimulates osteoclast differentiation [46].

The RANK/RANKL/OPG system is targeted by several hormones and inflammatory cytokines which can participate in the bone loss associated with inflammatory diseases, including obesity and diabetes. Indeed, levels of OPG are associated with fat mass [47] and atherosclerosis parameters in diabetes [47–49], while soluble RANKL have been shown to predict T2D in humans and to participate in the genesis of insulin resistance in this disease (see Section 5.6) [50].

## 4. Bone-Fat Interaction

Although an impaired balance between adipogenesis and osteogenesis may alter the number of bone forming cells, fat interferes with bone homeostasis trough several additional factors, which are often difficult to discern.

Epidemiological evidences have shown a positive correlation between bone mass and BMI suggesting fat to be protective against bone loss [51–53]. Notably, weight loss can increase bone turnover and decrease BMD [54–56], the latter effect partly mediated by increased levels of the WNT antagonist sclerostin [57]. Epidemiological studies have also suggested that weight loss-induced bone loss increases the risk for osteoporotic fractures in older adults [58, 59]. A main factor driving bone loss induced by weight loss is the reduction in mechanical stress on the weight-bearing skeleton mediated by changes in local bone factors (e.g., prostaglandins) and the mechanostat [60, 61], which is thought to be the osteocyte. Interestingly, in a recent trial on older obese adults, it was found that the addition of exercise training to weight-loss therapy prevented weight-loss-induced increase in bone turnover and attenuates weight-loss-induced reduction in hip BMD [62, 63], together with a maintenance of muscle strength [64] and lean mass [62]. The positive effect on BMD was shown despite weight-loss-induced decrease in bone-active hormones such as estradiol and leptin [63] and prevented the weight loss-induced increase in sclerostin levels [57].

However, when mechanical loading effect of total body weight is statistically removed, the association between BMI and bone mass becomes negative [65]. This is consistent with the recent evidence that obese people may have an increased risk of osteoporotic and hip fractures which is independent of BMD [66, 67], suggesting an independent effect of obesity and factors related to this disease on fracture risk. Fat is a source of a number of biologically active molecules that regulate metabolic homeostasis but also interact with bone metabolism [2].

### 4.1. Adipokines

#### 4.1.1. Leptin

It is produced primarily in the adipocytes of white adipose tissue, but it is also expressed in bone marrow adipocytes and osteoblastic cells. Leptin is involved in appetite and weight regulation and affects osteoblast proliferation and differentiation* in vitro* [68–70] and osteoclasts [68, 71, 72]. Leptin receptors are expressed in hypothalamus where their activation suppresses appetite. This hormone has also a peripheral action by targeting metabolically active cells such as insulin producing *β*-cells, osteoblasts, chondrocytes, and bone marrow stromal cells. The effect of leptin on bone metabolism is complex and data on rodents have yielded contradictory results. Mice deficient in either leptin (*ob*/*ob*) or the leptin receptor (*db*/*db*) displayed low trabecular bone volume and BMD in femur and tibia [73, 74]. Leptin deficiency was associated with an increase in the number and size of adipocytes in the femoral marrow [73], supporting* in vitro* data showing that this adipokine stimulated osteoblastogenesis while suppressing adipogenesis [69]. Leptin administration may improve bone formation and BMD in leptin-deficient mice but this effect is not evident when leptin levels are normal. Interestingly, leptin prevented bone marrow adiposity in T1D mice although it did not improve bone loss in this model [40]. On the other hand, Karsenty lab showed that leptin-deficient* ob/ob* mice exhibit increased vertebral bone mass [75]. Selective deletion of leptin receptor in osteoblast did not affect bone mass [76], while hypothalamic deletion of leptin receptor leads to increased bone mass that was reverted after intracerebroventricular infusion of leptin [75]. Taken together, these studies suggest that leptin has a direct effect on osteoblasts and bone marrow stromal cells but is also part of a very complex mechanism that regulates bone mass through a hypothalamic relay. Centrally, leptin inhibits bone formation, while peripherally it can decrease bone resorption and RANKL activity and increase formation enhancing the commitment of marrow-derived MSC to osteoblasts rather than adipocytes. Clinical studies also have provided conflicting evidences. According to some studies, but not all, leptin appears to be positively correlated with BMD [77]. The higher correlation is showed in postmenopausal women [77]. Women with vertebral fractures have significantly lower plasma leptin levels but not fat mass percentage [78], and increased leptin levels have been suggested to be protective against nontraumatic fractures independent of body weight [79]. Yet, other studies have found no relationship of leptin with either BMD or fractures [80, 81]. Thus, in summary, the role of leptin in clinical bone disease states is complex and needs clarification.

#### 4.1.2. Adiponectin

Exclusively produced by fat tissue, adiponectin circulates in much higher concentrations than other adipokines. In contrast to leptin, adiponectin is negatively correlated with visceral fat mass and BMI in humans, and low levels are described in patients affected by diabetes or myocardial infarction [82–85]. Adiponectin is structurally similar to TNF and RANKL [85].* In vitro* studies on the effect of adiponectin on bone cells yielded contradictory results. The majority of available data, however, suggest that adiponectin has an anabolic effect on osteoblasts and inhibits osteoclastogenesis, likely independently of RANKL and OPG [86, 87]. These actions would be expected to result in a positive effect of adiponectin on bone mass* in vivo*. In contrast, animal studies have found that adiponectin knock-out mice have increased both bone mass and trabecular number and lower bone fragility [86] and clinical results relative to the effect of adiponectin on BMD have been conflicting in humans. Some studies have reported an inverse association between serum adiponectin and BMD [88, 89], while others failed to detect any relationship in middle-aged men or women [90–93]. Among the most robust studies, that by Richards on 1,735 nondiabetic women found a strong negative correlation with BMD in postmenopausal women but not in the premenopausal ones, demonstrating the importance of menopausal status [94]. Similar results were recently described by Michaëlsson et al. [88] in a large cohort of elderly subjects and by Araneta et al. [95]. An inverse, but not statistically significant relationship was described also by Gonnelli et al., who studied elderly Italian men [93]. Recently, using more robust techniques that take into account bone geometry, such as peripheral quantitative computed tomography (pQCT), it was shown that adiponectin was inversely associated with bone mass in women but not in men [96]. A recent study have shown that, despite a positive association between BMI and BMD, a higher fat mass and lower lean mass were correlated with lower BMD in elderly adults. Inflammatory status (IL-6 and hs-CRP) and levels of adipokines leptin and adiponectin increased with increasing fat mass. In this study, adiponectin was the principal mediator of the apparent negative effect of fat mass on BMD [97]. Thus, in summary, the main body of evidence albeit not definitive appears to favor an inverse relationship.

#### 4.1.3. Resistin

It is a recently discovered adipocyte-secreted factor [98]. Resistin has rarely been found to be produced by fat tissue [99]. It is expressed by bone marrow and produced by peripheral mononuclear cells as an inflammatory cytokine [100]. Resistin is involved in the atherogenic process and serum levels are higher in diabetic and obese patients [98, 101, 102]. Recent studies observed that resistin can influence bone remodeling. This adipokine is expressed by MSC and promotes both osteoblast and osteoclast differentiation [103]. The effect of resistin on BMD is not clear, although a small inverse relationship between serum resistin and lumbar spine BMD in adult men has been reported [92] and higher resistin levels have been related to low total and cortical bone density, measured by CT, in older age [104].

### 4.2. Inflammatory Cytokines

Adipose tissue is an important “factory” of cytokines like interleukin- (IL-) 1, IL-6, and TNF that have been associated with bone loss. Levels of these cytokines are elevated in obesity and T2D and are directly related to bone fat and insulin resistance. They are also increased in patients with T1D as a result of the autoimmune activation in this disease.

#### 4.2.1. IL6

One-third of the circulating levels of IL-6 is produced by adipocytes and adipose tissue matrix [105]. This is consistent with the evidence that serum IL-6 is increased in overweight and obese individuals [106, 107]. IL-6 may affect glucose homeostasis and energy expenditure either directly or indirectly by acting on adipocytes, hepatocytes, skeletal muscle, and pancreatic *β*-cells [108]. T2D and obesity have been associated with a genetic polymorphism of IL-6 (−174 G/C) [109], supporting that level of expression of this molecule may influence metabolic homeostasis. High IL-6 levels have been associated with hyperlipidemia, hyperglycemia, and insulin resistance [110]. In contrast, intermittent exposure to IL-6 induces positive effect on blood glucose and energy expenditure [108] and administration in the central nervous system increases energy expenditure and decreases body fat in rats [110]. Similarly, the relationship between bone and IL-6 is ambivalent. As other inflammatory cytokines, IL-6 stimulates osteoclastogenesis [111] but may have also opposite effect by stimulating indirectly osteoblast proliferation or differentiation [112, 113].

#### 4.2.2. TNF

The NF-*κ*B pathway is the effector of TNF through the TNF receptor 1 (TNFR1) [114], expressed by macrophages and osteoclasts precursors. In the bone, TNF stimulates osteoclastogenesis enhancing expression of RANKL in several target cells including osteoblasts [115]. This promotes osteoclasts differentiations indirectly but also blocking osteoclasts apoptosis by acting via the mTOR/S6 kinase [116]. The main result is the increased lifespan of osteoclasts in proinflammatory environment [115]. TNF may also inhibit bone formation. In fact, a number of* in vitro* evidences have shown that high TNF levels can block both differentiation and proliferation of osteoblasts and their progenitors. NF-*κ*B signaling transduction, enhanced by TNF, is a potent inhibitor of osteoblast differentiation and activity. Some of these effects seem to be mediated by reduced Runx2 expression [117, 118] and also by the activation of the MAPK cascade [119]. Expression of osterix, a critical regulator of the early osteoblastic differentiation, is also inhibited [120]. Recently, it has been suggested that TNF can inhibit the Wnt *β*-catenin pathway by upregulation of Wnt inhibitors Dkk-1 [121] and sclerostin [122]. Notably, both TNF and sclerostin are increased in obesity and diabetes. Consistent with these findings, recent clinical studies have shown that patients on anti-TNF treatments have a significant decrease in bone resorption and osteoclastic activity [123].

### 4.3. Oxidative Stress

Obesity and diabetes are associated with increased oxidative stress [124, 125]. A low grade inflammation present in obesity and the abnormal activation of resident macrophages in the adipose tissues increase the levels or reactive oxygen species (ROS). A main source of ROS is the increased exposure of target tissue to inflammatory cytokines such as IL-1, TNF, and IL-6 which are increased in obesity. ROS may also directly regulate the activity of transcription factors, such as NF-*κ*B, thus controlling proinflammatory genes expression [126]. Moreover, dysglycemia frequently observed in obesity is associated with increased release of ROS by enhanced NADPH oxidase activity [124]. ROS have important direct effects on the differentiation and survival of osteoclasts, osteoblasts, and osteocytes [127, 128]. ROS disrupt the Wnt signaling pathway by altering the activity of FoxO transcription factors; under the ROS stimulus, these factors can decoy *β*-catenin preventing its binding to target genes necessary for osteoblast differentiation [129].

ROS have also an important action on the immune system and may indirectly promote osteoclastogenesis by altering the immunoskeletal interface. Superoxide upregulates the costimulatory molecules CD80 and CD86 [130]. CD80 promotes antigen-dependent T-cell activation and hence production of TNF by T cells. Another toxic effect of ROS is the induction of changes within the structure of protein. It has been suggested that this modifications can involve collagenous proteins and therefore potentially alter their properties.

### 4.4. Other Factors

#### 4.4.1. Sex Hormones

The adipose tissue can contribute significantly to the circulating pool of estrogens. Aromatase expression in adipose tissue primarily accounts for the peripheral formation of estrogen and increases as a function of body weight and advancing age [131]. The positive effect of estrogen on bone is obvious and exemplified by the fact that estrogen deficiency is the main cause of bone loss in postmenopausal women. According to* in vitro* studies, estrogens have a proosteogenic effect while preventing adipogenic differentiation of bone marrow stromal cells [132]. This is consistent with evidence that estrogen deficiency is associated with marrow adiposity in postmenopausal women [132] and estrogen administration can reverse marrow adiposity in the ovariectomised rat model [133–135]. Estrogen deficiency has been associated with a decrease in SIRT1 [134], a longevity factor previously associated with increased expression of the osteogenic factor Runx2 in MSC [136]. Estrogen deficiency leads also to increased oxidative stress into bone tissue which is supposed to negatively affect the balance between osteogenesis and adipogenesis [127]. Interestingly, estrogen replacement therapy in postmenopausal women [137] and elderly men have been associated with reduced levels of the WNT antagonist sclerostin [138, 139]. Oxidative metabolism of estrogen is another important determinant of postmenopausal bone loss [140, 141], bone mineral density in men [142], and body composition. Observational studies support an association between estrogen metabolism and BMI [143], suggesting that obesity is associated with significant decreases in hydroxylation of estrone at C-2. This results in reduced production of less active or inactive estrogenic metabolites, which can possibly sustain bone mass in obesity. Indeed, a recent study showed that, in postmenopausal women, an increase in the metabolism of estrogen towards the inactive metabolites is associated with lower body fat and higher lean mass than those with predominance of the metabolism towards the active metabolites [144].

While both estrogen and testosterone are important in bone health in both sexes, estrogen is the predominant sex hormone in females and testosterone in males. Increased aromatase activity in the excessive fat tissue may also lead to low testosterone levels in obese or T2D males. Approximately one-third of T2D males are testosterone deficient [145]. An even greater proportion of men who are both diabetic and obese experience testosterone deficiency, and the likelihood of testosterone deficiency increases as T2D progresses or worsens. Testosterone has been showen to prevent osteoclastogenesis in a way that is osteoblasts-dependent [146].* In vitro* data showed that exposure of human adipose-derived stem cells (hADSC) to testosterone or dihydrotestosterone in adipogenesis-inducing medium impaired lipid acquisition and decreased PPAR*γ*, C/EBP*α*, and C/EBP*β* gene expression [147]; another study found that such an effect may involve the WNT signaling pathway through the formation of a complex between the androgen, *β*-catenin, and the related transcription factor TCF4, thus involving the WNT signaling pathway [148]. Moreover, testosterone can prevent* in vitro *rosiglitazone-induced adipogenesis of MSC [149].

#### 4.4.2. Amylin

It belongs to calcitonin family and it is secreted with insulin. Amylin has central and peripheral effect, inducing satiety and gastric empty and reducing body weight and fat [150, 151]. Obese people have higher blood levels of amylin and reduced sensitivity to its action [150]. In the skeleton, amylin may stimulate osteoblast proliferation [152] and high serum levels have been shown to correlate with high bone mass. Amylin osteogenic actions may present different efficacy depending on the diabetic status. For example, amylin treatment in streptozotocin-induced diabetic rats increased bone volume and osteocalcin (OCN) levels but was not able to ameliorate diabetic osteopenia [153]. More recently, Gutiérrez-Rojas et al. showed that in rats with streptozotocin-induced T2D amylin increased osteoblast number and OCN expression in long bone and normalized trabecular structure; on the contrary, insulin resistant rats treated with amylin did not present any apparent osteogenic effect in the femur, although both OCN and OPG/RANKL ratio were increased in the tibia. These findings demonstrate a different osteogenic efficacy of amylin in two diabetic settings [154].

#### 4.4.3. Ghrelin

Ghrelin is a polypeptide mainly secreted from neuroendocrine cells of the fundus of the stomach and in smaller amounts from renal, pituitary, and hypothalamus cells [155, 156]. Ghrelin is believed to play an important role in energy balance and in food intake. Its serum concentration is inversely associated to BMI and increased in diet-induced weight loss [155, 156]. Recent studies showed that ghrelin may be produced by osteoblasts, and ghrelin receptors were detected in both rat and human bone cells [157]. Although ghrelin has a positive effect on osteoblast proliferation and differentiation [158–161],* in vivo* studies on animals have been contradictory showing either no association with bone mass in mice [158] or a positive effect in rats with increased osteoblast-like cells number, expression of osteoblast differentiation markers, and BMD [157].

The InChianti study has shown that serum ghrelin is positively correlated with trabecular BMD, measured by pQCT, in a cohort of 401 elderly healthy Italian women [162]. Similarly, Gonnelli et al. described a significant, positive effect of ghrelin on femoral neck BMD in elderly men [93]. On the contrary, using dual-energy X-ray absorptiometry (DEXA) measurement, no significant effects of ghrelin on BMD at any bone sites were found in 80 Korean middle aged males [92] or in the Rancho Bernardo cohort after adjusting for BMI and age [163].

#### 4.4.4. Hydroxyl Steroid Dehydrogenase

Glucocorticoids serum level is connected to obesity and bone metabolism. 11-*β*-hydroxysteroid dehydrogenase (11b-HSD) 1, which converts inactive cortisone into active cortisol, is involved in adipocyte differentiation and obesity [164]. In fact 11b-HSD1 is expressed in adipocyte, while 11b-HSD2, which inactivates glucocorticoids, is not expressed [165]. 11b-HSD2 knock-out mice are protected from obesity with low levels of glucocorticoid [165, 166], and they are resistant to hyperglycemia induced by stress or high fat feeding [167]. In obese humans serum 11b-HSD is elevated [165, 168, 169] and more active compared to nonobese individuals. 11b-HSD1 is also expressed by osteoblasts and osteoclasts [170, 171]. The expression of osteoblastic 11b-HSD1 determines the synthesis of active glucocorticoids. This has consequent effects on osteoblast proliferation and differentiation and the risk of induced osteoporosis increases with age and depends on autocrine actions of the enzyme 11b-HSD1 [172].

## 5. Diabetes-Bone Interaction

Obesity is prevalent in patients with diabetes. Although diabetes-induced bone loss is partially dependent on obesity related factors, there are other diabetes-specific elements that can increase further the deleterious effect on bone metabolism. Among these factors, hyperglycemia is the hallmark of diabetes which is ultimately associated with chronic complications. These complications are common both in T1D and T2D and can impact bone health directly or indirectly by increasing the risk of falls. Finally, T1D and T2D differ by the presence of some more specific pathophysiological elements, such as insulin deficiency in T1D compared with insulin-resistance or loss of incretin effect in T2D. All these factors can impact differently bone metabolism.

### 5.1. Impaired Calcium Balance and Vitamin D Deficiency

High blood glucose may increase urinary calcium excretion and generate several interactions with the parathyroid hormone (PTH)/vitamin D axis [173–175]. Increased calcium loss is associated with hypocalcemia and suppression of PTH secretion. Conversely, improvement of blood glucose control may reduce calcium and phosphate urinary excretion and 1,25(OH)_2_ vitamin D_3_ (1,25(OH)_2_D_3_) levels and increase phosphate levels but without improving serum calcium or PTH [176]. Numerous studies have shown that both patients with T1D and T2D have impaired vitamin D status. A cross-sectional study on 5,677 patients with T2D and impaired glucose tolerance had significantly lower 25OH vitamin D (25OHD) levels compared with controls [177]. Obesity itself is also associated with altered vitamin D metabolism and impaired vitamin D status, which is likely due to the decreased bioavailability of vitamin D because of its deposition in body fat compartments [178]. Indeed, although higher levels of 25OHD have been associated with 13% reduced risk of diabetes, this effect was attenuated after adjustment for BMI [179]. Vitamin D supplementation aimed at raising 25OHD levels above 30 ng/mL had no effect on insulin secretion, insulin sensitivity, or the development of diabetes compared with placebo [180]. Similarly it is documented that new-onset T1D patients have reduced levels of 1,25(OH)_2_D and 25OHD compared with healthy controls [181]. Here, a causative role of vitamin D deficiency in diabetes has been suggested although not proven by intervention trials thus far. Moreover, vitamin D supplementation in young patients with new-onset T1D did not improve markers of bone turnover [182]. However, no trials of vitamin D supplementation on BMD or fractures are available in patients with diabetes. A major complication of diabetes is renal impairment with osteodystrophy. Here disturbances of calcium, phosphate, FGF-23, and Vitamin D physiology may have a major impact on the bone health of diabetic patients. This topic is beyond the scope of this paper.

### 5.2. Hyperglycemia and Oxidative Stress

High blood glucose induces formation of advanced glycation end-products (AGE), with negative effects on structural proteins such as type I collagen, the main bone matrix protein. This nonenzymatic glycosylation is a multistep process. Glucose leads to the formation of a Schiff's base which is further degraded to a class of intermediate products containing highly reacting dicarbonyls. Reaction of these carbonyls with the NH_2_ side chain containing amino acids (arginine, lysine, and hydroxylysine) leads to formation of irreversible AGE compounds such as pentosidine and N^*ɛ*^-carboxymethyllysine (CML) [183]. AGEs may have damaging effects on collagens by forming irreversible cross-links between the fibers in the triple helix [184]. AGE involving collagen and other structural or circulating proteins are a source of ROS that can further induce structural changes by means of posttranslational modifications [185]. Since collagen is a structural protein with relatively slow turnover, the irreversible changes induced by glucose and ROS are retained within the tissues. These changes are linked to reduced strength and impaired biomechanical properties of both cancellous and cortical bone [186]. This is consistent with the clinical evidences that increased levels of pentosidine in patients with diabetes are associated with a more frequent history of spine fractures, independently of BMD measured by DEXA. It has been shown that high urinary pentosidine levels were associated with a 42% increase of clinical fractures incidence in patients with diabetes compared with controls [187]. Similarly, serum levels of pentosidine were higher in diabetics patients who experienced vertebral fractures [188].

AGE may also reduce bone strength by impairing bone formation. It has been shown that AGE disturb osteoblast function [189] and attachment to collagen matrix [190] and interfere with their normal development [191]. Circulating AGE can also bind specific receptors called RAGE located on osteoblasts and immune cells [192, 193]. These receptors enhance production of inflammatory cytokines and ROS, feeding a vicious circle of chronic inflammation [192] and increased bone resorption [193]. Hyperglycemia can be “toxic” to osteoblasts themselves. Botolin et al. have shown that acute (24 h) hyperglycemia and its associated hyperosmolality suppress expression of genes involved in osteoblast maturation [194] including OCN [195, 196]. Similarly, chronic hyperglycemia downregulates OCN expression [197] and calcium uptake in osteoblast cultures [198], while increasing PPAR-*γ*2 expression [199]. In mice, blood glucose levels are positively related with bone marrow induced osteoblast death and negatively related with OCN expression in bone [200].

Hyperglycemia and oxidative stress may also affect MSC differentiation. There is evidence that adipogenesis may prevail over bone formation when adipose tissue-and muscle-derived stem cells are exposed to high glucose concentration. Culturing MSC with high glucose media induced expression of adipogenesis markers such as PPAR-*γ*2, GLUT4, and adiponectin but reduced osteogenic (OCN, osteopontin, osteonectin) or chondrogenic (type II collagen) markers. The adipogenic shift was mediated by ROS through enhanced signaling by PKC-*β* [201]. Diabetes is also linked to a generalized damage of blood vessels wall, which results in chronic micro-and macrovascular complications. Oxygen tension within the marrow microenvironment is physiologically lower than other tissues and the diabetes status may further alter cellular homeostasis. Indeed, some authors have shown reduced differentiation of MSC toward either adipose or osteoblast phenotype [202, 203], while others suggested an increased differentiation of MSC under hypoxia conditions [204, 205]. Finally, hyperglycemia-induced acidosis may enhance bone resorption and impair bone quality [128].

### 5.3. Insulin Deficiency

Clinical,* in vivo,* and* in vitro* studies suggest that insulin exerts a bone anabolic effect on osteoblasts [206]. In humans, insulin deficiency was associated with reduced bone mass in a study of 62 new-onset T1D subjects evaluated before insulin therapy. Treatment with insulin after 7 years improved substantially bone mass and markers of bone turnover [207]. On the other hand, hyperinsulinemia present in patients with T2D may contribute partially to the higher BMD [208].

Anabolic action of insulin in osteoblasts seems to be mediated by increased Runx2 activity through suppression of its inhibitor Twist2 [209]. Runx2 is a major factor involved in osteoblast differentiation and proliferation, and its expression is actually impaired in models of T1D [210]. In mice lacking the insulin receptor in their osteoblasts bone formation is impaired and associated with a reduced number of osteoblasts and reduced trabecular bone volume [209]. These mice have also a decreased osteoclast activity as showed by reduced osteoclast erosion depth and serum levels of cross-linked C-telopeptide (CTX) [209]. The insulin receptor signals through four insulin receptor substrates molecules (IRS-1 to IRS-4). Mice lacking these substrates showed abnormal bone phenotype [211, 212]. IRS-1 deficient mice showed impaired bone healing which was restored after its reexpression in the fracture site [213], and IRS-2 knock-out mice had reduced bone formation over resorption [211]. Part of the insulin signaling through the IRS may be mediated by the IGF-I [214]. Levels and/or action of IGF-I and PTH, another bone anabolic hormone, are also impaired in insulin deficiency conditions [153, 214, 215].

Moreover, studies on T1D animal models confirm that insulin deficiency adversely affects skeletal homeostasis. Streptozotocin-induced diabetic mice and nonobese diabetic (NOD) mice have low-turnover osteopenia [216] associated with a disruption in osteoblast [153, 217] and its multipotential mesenchymal precursor [194, 199]. Analysis of gene expression in mesenchymal cells from NOD mice bones demonstrated a switch from genes associated with a mature osteoblast phenotype to genes associated with an adipocyte phenotype. PPAR-*γ*2 and aP2, known markers of adipocyte differentiation and maturation, were raised in diabetic NOD mice, together with increased number of adipocytes. In contrast, expression of OCN was significantly decreased in diabetic NOD mice. Suppression of osteoblast maturation, demonstrated by lowered OCN mRNA levels, was correlated with decreased bone density in both NOD and streptozotocin-treated mice [194, 199, 216].

### 5.4. Loss of Incretin Effect (Gut-Hormones Interaction)

The incretin effect is the increase of glucose stimulated insulin secretion resulting from the release of intestinally derived peptides in response to glucose or nutrients in the gut. The incretin effect depends primarily on two peptides, glucose-dependent insulinotropic polypeptide (GIP) and glucagon-like peptide 1 (GLP-1). GIP and GLP-1 have short half-life and are rapidly degraded by dipeptidyl peptidase-4 (DPP-4). This pathway is attenuated in T2D [218] and the therapeutic target of drugs commonly used in T2D such as GLP-1 receptor analogues, which are resistant to DPP-4 degradation, and inhibitors of DPP-4, which extend the half-life or the native incretins [219].

GLP-1 receptors are expressed on bone marrow stromal cells and immature osteoblasts [220, 221], and GLP-1 stimulates proliferation of MSC and inhibits differentiation to adipocytes [221]. GLP-1 receptor knockout mice have decreased cortical bone mass due to increased osteoclast number and activity [222], impaired mechanical and material properties [223], and decreased calcitonin secretion from thyroid C cells [222]. In fact, GLP-1 receptors are also expressed on thyroid C cells and therefore increase the secretion of calcitonin [224], which could contribute to the postprandial decrease in bone resorption.

In animal models, administration of GLP-1 (3 days) increases bone formation in normal rats and rats with streptozotocin-induced diabetes or fructose-induced insulin resistance, suggesting an insulin-independent action [225]. The diabetic and insulin resistant rats also demonstrated improvements in trabecular bone mass and microarchitecture. Also, 3 days of continuous administration of the GLP-1 analogue exenatide increased markers of bone formation and may improve microarchitecture in normal rats and rats with streptozotocin-induced diabetes or fructose-induced insulin resistance [225]. The positive effects of GLP-1 treatment on trabecular bone mass and microarchitecture may be likely mediated by a positive effect on bone formation and on OPG/RANKL ratio [226].

Effects on bone formation may be mediated by interference with WNT signalling, considering that incretin treatment in type 2 diabetic rats has been reported to lower serum levels of sclerostin and increasing serum levels of OCN [227]. Wnt signaling and its elements are important for both the production and function of the incretin hormones [228]. Grant et al. showed that the TCF7L2 variant 7903146, the main T2D associated gene [229], modifies the effect of incretins on insulin secretion [230]. On the other hand, Wnt signalling cascade increased expression of proglucagon gene* gcg* and* gip *mRNA [231]. García-Martínez and colleagues showed that Wnt/*β*-catenin signaling or lithium, which mimics the Wnt signaling, enhanced GIP production by enteroendocrine cells through a conserved TCF binding site within the proximal region of the* gip *promoter [231].

### 5.5. Sclerostin and WNT Signaling Pathway

Recent studies have suggested that part of the negative effect of diabetes on bone quality may be mediated by disturbances in the WNT signaling pathways. The glycoprotein sclerostin acts as an antagonist for the WNT/*β*-catenin canonical signaling pathway; this molecule is produced by osteocytes and exerts its inhibitory effect by binding to LRP-5 and/or 6 on osteoblasts. In mice with streptozotocin-induced T1D, WNT signaling is suppressed but also sclerostin is downregulated [232]. In these mice an increased osteocyte apoptosis and lower total and nuclear *β*-catenin staining was shown [232]. In contrast, T2D rats presented enhanced expression of Dkk-1 and SOST/sclerostin gene; SOST overexpression related to increased mRNA levels of the WNT activator LRP-5 [225]. Clinical studies have also showed that levels of sclerostin are higher in patients with T2D compared with control subjects [233], inversely related with bone turnover markers [233, 234], and positively associated with spine and hip BMD [233]. In both diabetic and healthy subjects, sclerostin levels were higher in males than females. In another study, sclerostin was higher in T2D than either healthy controls or T1D [235]. These findings suggested that elevated sclerostin levels may influence bone fragility and bone quality associated with T2D. Indeed, there is increasing evidence that sclerostin levels may predict the risk of hip and other osteoporotic fractures both in postmenopausal women [236, 237] and T2D [238, 239] as well as that sclerostin antibody targeting can ameliorate diabetic bone loss in rodents [240]. However, it should be noted that the assay for sclerostin is relatively new and has not been universally standardized [241] and this may have affected the results from different studies.

### 5.6. RANKL System and Insulin Resistance

RANKL is a member of the TNF superfamily and, after binding to its cognate receptor RANK, acts as a potent stimulator of NF-*κ*B. There are evidences supporting a role of this system in diabetes and associated diseases [242]. Both liver tissues [243] and *β*-cells express RANKL and RANK [50]. Moreover, levels of OPG, which competes for RANK/RANKL interaction, are elevated in T2D, especially in those with poor glycemic control, and relates with fat mass and atherosclerosis parameters [47–49, 244]. Recently, Kiechl et al. showed that increased levels of soluble RANKL are associated with the development of diabetes in 844 subjects from the Brunek study (OR = 3.37; 95%CI: 1.63–6.97). Besides the epidemiologic finding, the authors showed that RANKL interacted with glucose homeostasis by acting on hepatocytes function and insulin resistance. Indeed, mice selectively lacking RANK in the hepatocyte (*Rank*LKO) were protected by high-fat diet induced insulin resistance and showed fasting glucose and insulin concentrations similar to those of* Rank*WT mice fed a normal-fat diet [50]. These data suggest the RANKL involvement in the pathogenesis of hepatic insulin resistance and T2D and provide a link between inflammation and disrupted glucose homeostasis.

### 5.7. Osteocalcin and Glucose Homeostasis

Not only does abnormal glucose homeostasis have deleterious effects on bone metabolism, but also the rate of bone turnover may in turn regulate glucose homeostasis. Recent research has showed that the osteoblast-derived protein OCN has endocrine effects, acting on islet cells stimulating *β*-cells proliferation and insulin secretion. Karsenty group has indicated that OCN-knockout mice display decreased *β*-cells proliferation, glucose intolerance, and insulin resistance. In* ex vivo *studies, when pancreatic *β*-cells isolated from wild-type mice were cocultured with wild-type osteoblasts or in the presence of supernatants from cultured osteoblasts, insulin secretion increased, suggesting the presence of an osteoblast-derived circulating factor that regulates *β*-cell function. Administration of OCN significantly decreased glycaemia and increased insulin secretion. Furthermore, OCN function was exerted through adiponectin-coculture of wild-type osteoblasts with adipocytes increased adiponectin expression and action [245, 246]. It is well known that insulin is involved in OCN production. More recently, insulin was also shown to be a key factor in regulating its bioactivity; OCN expression is mediated by suppression of* Esp* signalling by insulin within the osteoblast [247]. According to animal studies, OCN is a prehormone which is active only in its undercarboxylated form (ucOCN) [245]. However, clinical studies did not show consistent difference between the two forms of OCN, and vitamin K administration, which is believed to increase the rate of OCN carboxylation, has been unexpectedly associated with improved insulin sensitivity [248–250]. OCN decarboxylation is a pH dependent mechanism permitted in presence of increased osteoclastic reabsorption under the insulin stimulus [247]. Recently, it has been shown that ucOCN can signal trough the specific receptor Gprc6a expressed on beta cells [251]. Animal studies have suggested that testis may be also intercalated into this fine loop whit OCN inducing testosterone production by the Leydig cells [252]. Clinical studies did not confirm that this finding where subjects administered a bisphosphonate for osteoporosis had very low OCN values but reports of diabetes induction or worsening have been reported from the trials.

A number of human studies have also explored the relationship between OCN and glucose homeostasis. OCN levels have been reported to be lower in T2D compared to healthy subjects [253], inversely related to body mass index, fat mass, and plasma glucose [254–257] but also to atherosclerosis and inflammatory parameters such as high sensitive C-reactive protein and IL-6 [258]. However, most of these studies were conducted in healthy subjects or T2D only and limited by the cross-sectional design. On the other hand, studies evaluating treatments or conditions which are able to change OCN levels have shown opposite results questioning the OCN-glucose relationship in humans. For example, alendronate therapy, which decrease OCN levels, was associated with reduced diabetes risk [259]; treatment with vitamin K, which reduces ucOCN/OCN rate, improved insulin resistance [248–250]; chronic hyperparathyroidism, which is characterized by increased OCN release, was associated with increased insulin resistance and impaired glucose regulation [260].

Less evidence is available for T1D. A recent study showed no association between OCN and *β*-cell function in subjects with new-onset T1D [182]. Although human studies in patients with T2D and in animals support a positive feedback between osteoblasts and *β*-cells, authors speculated that, in a condition of continuous autoimmune damage against *β*-cell such as in T1D, the OCN may be ineffective on controlling *β*-cell function. On the other hand, Thraikill et al. reported a positive effect of OCN on endogenous insulin production (assessed by authors as C-peptide/glucose ratio) [261] in subjects with long standing disease.

Thus at this stage it appears that the relationship of OCN and glucose homeostasis appears to be most robust from* in vitro* and animal studies but its role in humans is less clear and requires further investigation.

## 6. Diabetes and Fractures in Clinical Practice

The occurrence of fractures in diabetes mellitus is increased but the evidence for fracture occurrence has been reported from cross-sectional observational cohorts which have inherent weaknesses. Most of the risks have largely been derived from surrogate markers, for example, bone turnover markers, bone histology, DEXA, and other imaging modalities, showing predominantly a bone quality defect. Randomized prospective trials designed to assess fracture risk specifically and/or treatment are not available. The section below will describe epidemiology of and clinical factors associated with fractures in both T1D and T2D.

### 6.1. Bone Turnover Markers

A recent study has demonstrated that in patients affected by T1D, OCN levels are 4 or 5 times lower than in control subjects, while in T2D patients OCN is 2 or 3 times lower than in controls [262]. Other studies have been performed to individuate a possible link between glucose balance and bone markers. In particular, it has been demonstrated that, both in T1D and in T2D, higher levels of HbA1c are associated with lower levels of OCN [262, 263]. Scientific evidences have demonstrated that Type 1 diabetic children present a severe reduction of OCN levels mainly during sexual maturation. This represents a very significant cause of the missed goal of age-related peak bone mass, which takes place generally between 18 and 30 years. Bone markers belonging to protein products of collagen breakdown, including CTX (bone resorption) or type 1 procollagen N-terminal peptides P1NP (bone formation), and the complex pathway directed by OPG and RANKL, seem to be associated negatively with glucose balance. Studies have shown that higher levels of HbA1c correspond to lower levels of bone apposition markers including lower levels of OCN and OPG [262].

A recent study by Rubin group [216] has identified an alteration in circulating osteogenic precursor cell (COP) in T2D. These circulating cells arrive at bone formation site through blood vessels and form osteoblast-like cells [206, 209]. COP is characterized using antibodies against OCN. Subjects with T2D have a decrease in circulating OCN-positive cells, while the same subjects show an increase of immature OCN-positive cells with early markers CD146 (a marker of bone cells progenitors) and CD34 (which identify cells that can increase osteoblast function), which consequently mean small pool of immature COP cells. Moreover T2D subjects with HbA1c higher than 7.9% had higher levels of these immature cells [216]. Although questions exist about the role of circulating OCN cells in bone formation, there is evidence that osteoblast progenitor cell maturation in T2D is inhibited. OCN+/CD146+ cells presented lower expression of the Runx2 master marker of osteoblast differentiation and increased markers of oxidative stress. In the same study, bone formation markers such as P1NP and OCN were significantly lower in T2D. Similarly, bone resorption marker serum CTX was lower in T2D, although the difference in tartrate-resistant acid phosphatase (TRAP)-5b levels was not significant, suggesting low bone turnover. Moreover, the differences in turnover markers correlated with blood glucose control as showed by an inverse relationship between HbA1c and bone markers P1NP, OCN, and CTX [216].

### 6.2. Histology

#### 6.2.1. Type 1 Diabetes

Microstructural defects or alterations in bone turnover may play a central role in diabetic osteopathy. Verhaeghe et al. [264] performed two studies on diabetic mice after 3 or 4 weeks after the onset of the disease. Serum OCN levels, osteoblast/osteoclast and osteoid surface percentages, and the daily mineral apposition rate were reduced in diabetic mice (mineral apposition rate in the tibia 1.0 ± 0.4 versus 5.6 ± 0.6 *μ*/day and vertebra 0.2 ± 0.1 versus 2.3 ± 0.2 *μ*/day), proving that osteoblast function is compromised with a consequent low bone turnover [264]. In the second study diabetic mice presented 25% less stiffness and strength in the femur than nondiabetic mice and a lower resistance to physical exercise. Finally, a 50% increased collagen cross-linking to the AGE Pentosidine was observed [265].

Armas et al. [210] compared bone histomorphometric and *μ*-CT results from iliac biopsies from 18 subjects with T1D on insulin treatment without complications and a good metabolic control. They found no differences in terms of histomorphometric or *μ*CT parameters between diabetics and controls. However, fractured patients had a trend for abnormalities in structural and dynamic variables, such as lower BT/TV%, suggesting defects in their skeletal microarchitecture [210].

In conclusion, data obtained by the described studies seem to suggest that in T1D low bone formation delays bone apposition during growth and increases bone resorption, with a worsening effect related to the duration of the disease and to the glycemic control.

#### 6.2.2. Type 2 Diabetes

Increased fracture risk in T2D could be related, among the others, to alterations in skeletal structure. Dobnig et al. [211] have investigated the effect of T2D on bone turnover in 583 T2D patients and 1,081 control subjects, while hip and other nonvertebral fractures were monitored over 2 years. Diabetic patients had significantly higher age-, weight-, and mobility score-adjusted calcaneal stiffness (*P* < 0.0001), radial speed of sound (*P* < 0.005), and phalangeal speed of sound (*P* < 0.05) revelations in comparison with controls. Serum PTH (−20.7%) and OCN levels (−22.3%) were significantly lower (both *P* < 0.0001) in T2D patients despite similar low serum 25OHD levels. However, a total of 110 hip fractures occurred during the observation period, with a similar hip fracture rate in controls and T2D (3.1 and 3.4 % per 100 patient years, resp.). Shu et al. [266] recruited 25 T2D women and 25 female control subjects and performed high-resolution peripheral quantitative computed tomography (HR-pQCT) and bone turnover markers. The results of the study showed that HR-pQCT did not differ among the two groups but both P1NP and OCN resulted lower in diabetic women than in controls (*P *≤ 0.005 and <0.001, resp.), suggesting that T2D could present a lower bone turnover regulation [266]. Okazaki et al. [176] performed a study on 78 T2D patients with a poor glycaemic control (HbA1c > 8%) and measured the serum bone markers at the baseline and after 3 weeks of glucose lowering treatment. Bone resorption markers were decreased at the beginning of the study while they were increased after the 3-week treatment, proving that a good glycaemic control influences bone turnover [176]. As stated above, COP cells have been recently identified and studied in osteoporotic patients. Rubin's group has correlated COP with bone histomorphometric structure and bone markers in T2D patients [216]. The results of the study showed reduced COP cells in T2D patients in comparison with control subjects. The bone formation markers P1NP and OCN were significantly lower in T2D (P1NP *P* < 0.01, OCN *P* < 0.03), as the bone resorption serum CTX (*P* < 0.01). Reduced histomorphometric indices of bone formation were observed in T2D subjects, including mineralizing surface (2.65 ± 1.9 versus 7.58 ± 2.4%, *P* < 0.02), bone formation rate (0.01 ± 0.1 versus 0.05 ± 0.2  microm^3^/um^2^× d, *P* < 0.02), and osteoblast surface (1.23 ± 0.9 versus 4.60 ± 2.5%, *P* < 0.03) [216]. Although questions exist about the role of circulating OCN cells in bone formation, evidence that osteoblast progenitor cell maturation is inhibited in T2D could lead to interventions targeting improved bone formation to enhance bone strength in the diabetic skeleton.

Burghardt et al. [267], using HR pQCT, showed that T2D is associated with impaired microarchitecture and biomechanics at the peripheral skeleton of elderly women. Diabetic women had 10% higher trabecular volumetric BMD adjacent to the cortex and 13.8% higher trabecular thickness in the tibia. Cortical porosity was increased in the radius while pore volume tended to be higher in the tibia suggesting impaired resistance to bending loads and inefficient redistribution of bone mass in diabetes.

### 6.3. Bone Density and Strength

#### 6.3.1. Type 1 Diabetes and BMD

It is not yet completely clear how BMD, osteoporosis, and the risk of fractures are related in T1D and T2D. Many, but not all, studies performed on T1D patients reported low BMD values at DEXA measurements [174] and focused their attention on the role of diabetic microvascular complications in the pathogenesis of osteoporosis. Insulin-dependent diabetic patients, in fact, show an approximately 10% decreased bone mineral content (BMC) a few years after clinical onset of diabetes [268]. However it seems that, in absence of diabetic microvascular complications, a further bone loss does not occur. Mathiassen et al. [268] studied 19 insulin-dependent diabetic patients and determined BMC with an interval of 11 years. At initial examination, no patient had diabetic microangiopathy, but at final examination 7 patients had developed diabetic microvascular complications. In comparison with gender- and age-matched controls, both subgroups showed significantly lower BMC at the initial examination, but, at final examination, BMC was significantly decreased in patients with microvascular complications than in patients without. Blood tests for bone metabolism showed a significantly increased fasting urinary excretion of calcium and hydroxyproline in patients with complications, but not in the group without complications, and there was a negative correlation between plasma OCN and HbA1C for all patients [268]. Forst et al. [269] found a 10% reduction of bone mineral density in the femoral neck (*P* < 0.01) and a 12% reduction in the distal radius (*P* < 0.001) compared with the control group. No significant difference was found in the lumbar spine. A link between decreased bone mineral density and diabetic neuropathy was observed for the femoral neck (*P* < 0.001), but not for the distal radius or axial skeleton, demonstrating that T1D microvascular complications may influence bone health [269]. Munoz-Torres et al. [270] performed a study on 94 patients affected by T1D and with disease duration ranging from 1 to 35 years. Diabetic patients showed reduced BMD in all sites and 19.1% met diagnostic criteria for osteoporosis. Diabetic complications were associated with lower BMD concluding that osteopenia and osteoporosis are a common complication of T1D and microvascular complications are a critical point in the progression of diabetic osteopenia [270].

#### 6.3.2. Type 2 Diabetes and BMD

Barrett et al. [271] found that diabetic men had similar BMD compared to those with normal glucose tolerance, whereas diabetic women had higher BMD at all sites. The increased bone density in diabetic women was unexplained by age, obesity, cigarette smoking, alcohol intake, regular physical activity, and the use of diuretics and estrogen. Older women with T2D or hyperglycemia had better BMD than women with normal glucose tolerance, independent of differences in obesity and many other risk factors. No differences in bone density by diabetic status were observed in men. In conclusion, it is possible that the sex differences may be explained by the greater androgenicity reported in women with hyperglycemic and hyperinsulinemic conditions [271]. Similar findings were found by Stolk et al. [208] with higher bone mass associated with higher glucose and postload insulin levels at all bone sites. In men, the mean age-adjusted BMD at the lumbar spine increased by 4.64 per mmol/L serum glucose (95%CI 1.46–7.82) and 0.35 per mU/L postload insulin (0.17–0.53). In women, these values were 6.88 (4.37–9.39) for glucose and 0.25 (0.11–0.39) for insulin (for all analyses: *P* < 0.01) [208]. Following the same hypothesis, Barrett et al. found that hyperinsulinemia could play an osteogenic role showing that each 10 microU/mL increase in fasting insulin was associated with a 0.57 g/cm^2^ increase in lumbar spine [271].

#### 6.3.3. Bone Strength

Bone strength may be reduced even in absence of changes in areal BMD because of geometric changes. In a case-control study, Petit et al. [272] examined the association between T2D and bone volumetric density, geometry, and estimates of bone strength at both tibia and radius. T2D patients had higher volumetric BMD (vBMD) and a smaller bone area, but no differences in estimated compressive bone strength at the distal trabecular bone regions. On the other hand, total bone area was smaller at the cortical bone midshaft sites resulting in lower bone bending strength despite a similar vBMD at these sites, suggesting that bone strength may be impaired in absence of vBMD changes.

Recently, Farr et al. [273] performed* in vivo *microindentation testing of the tibia to directly measure bone mineral strength in 60 postmenopausal women including 30 patients diagnosed with T2D for >10 yrs. Bone mineral strength was significantly lower in patients with T2D than controls and porosity tended to be increased in these patients despite no significant changes in other bone microarchitecture parameters. Glucose control was inversely related with strength and bone turnover markers were reduced [273]. Using HR pQCT, Patsch et al. [274] showed that fracturing diabetic patients had higher intracortical pore volume, relative porosity, and endocortical bone surface than diabetics without fractures. Relative porosity at the distal radius was 4.7-fold higher in fracturing diabetics compared with nonfracturing patients. Similarly, ultradistal tibia had more porosity and trabecular heterogeneity was higher in fractured diabetic patients [274]. On the other hand, nondiabetic fractured subjects and healthy controls only differed in a slight increase in pore volume. Similarly, using MRI at the distal radius, Pritchard et al. [275] found that women with T2D had larger holes within the trabecular bone network than women without T2D.

### 6.4. Predicting the Risk of Fractures

Even the FRAX (fractures risk assessment tool), an algorithm adopted by the WHO to assess the risk of fractures, does not seem useful in T2D patients [186]. In fact, Schwartz et al. have indicated that fracture risk was higher for a given T-score and age or for a given FRAX score [276]. Like BMD, FRAX score is only partially effective to predict hip and nonspine fracture risk in T2D patients.

A novel bone-state parameter is the trabecular bone score (TBS). It is a texture parameter that evaluates pixel gray-level variations in the spine DEXA image and is related to bone microarchitecture and fracture risk, independent of BMD. A positive correlation between lumbar spine TBS and skeletal deterioration in postmenopausal women with diabetes has been demonstrated, while in the same cases BMD is greater [277]. Data suggest that a TBS and BMD correlation could improve fractures prediction [278–281].

Therefore assessment of fracture risk in diabetics cannot be based only on traditional risk factors and commonly used algorithms and new predicting factors are needed.

### 6.5. Fractures in Type 1 Diabetes

Fractures risk is significantly higher in T1D when compared to the general population as well as to patients with T2D [282]. Most of the studies have focused on hip fractures finding a higher relative risk (RR) ranging between RR 1.7 and 12.3 [283]. Fractures at the spine and proximal humerus also were moderately increased [284, 285].

No gender differences were found although the small number of studies assessing this relationship does not allow a definite conclusion. A meta-analysis of 5 cohort studies showed that T1D was associated with an overall RR of 8.9 (95%CI: 7.1–11.2) [283]. In the large prospective Nurses' Health Study the incidence of hip fractures was reported as 383 per 100,000, a result 6-fold higher than the overall incidence of hip fracture in this population and 2.5-fold higher than in T2D [282].

Ivers et al. reported a higher risk of fractures in patients presenting retinopathy [286], while Strotmeyer et al. have shown that falls, lower performance state, neuropathy, and stroke were more frequent in fractured patients than in those without fractures [287]. Miao et al. reported a strong correlation between fracture risk and all types of complications, in particular with a lower BMD in patients with neuropathy and nephropathy than in patients without these complications [288].

### 6.6. Fractures in Type 2 Diabetes

#### 6.6.1. Hip Fractures

Hip fractures contribute the most to the fracture risk seen in T2D [289–291]. This risk appears to be slightly higher in men compared with women [283, 292] and in black compared to white women [293]. The Nurses' Health Study showed that the incidence of hip fractures in women with T2D was 153 per 100,000 subjects compared with 63 per 100,000 [282]. The incidence was even higher in those women treated with insulin (209 per 100,000) [282]. Two large meta-analyses that assessed studies involving 1.3 million subjects confirmed that patients with T2D are at increased risk of hip fractures, with a RR of 1.7 (95%CI: 1.3–2.2) [283] and 1.38 (95%CI: 1.25–1.53) [294], respectively. The association increased further when the analysis was restricted to 4 cohorts with more than 10 years of follow-up, RR 2.7 (1.7–4.4) [283].

#### 6.6.2. Vertebral Fractures

There is very little data available regarding vertebral fracture risk in T2D. Three studies have independently showed that risk for vertebral fractures is similar to nondiabetics [284, 287, 293]. However, a recent Japanese study found that diabetes was associated with increased risk in women (OR = 1.9; 95%CI: 1.11–3.12) and men (OR = 4.7; 95%CI: 2.19–10.20) [295]. Contrary to what shown in controls, age and BMD did not predict fractures in T2D patients.

#### 6.6.3. Extremity Fractures

Fractures of wrist [296] and foot [289, 293] also seem to be more frequent in T2D. These data were confirmed in a recent meta-analysis and appeared to be true only in those patients treated with oral hypoglycemic agents or insulin [283].

#### 6.6.4. Atypical Femur Fractures

Atypical low-energy subtrochanteric and diaphyseal fractures have been reported as a possible adverse event associated with bisphosphonate therapy. A recent analysis of the Study of Osteoporotic Fractures (SOF) has shown that history of diabetes was the strongest independent predictor of this type of femur fracture (HR = 3.25; 95%CI: 1.55, 6.82) [297].

### 6.7. Relationship with Disease Duration and Complications

In some studies, T2D was not associated with fractures [211, 298, 299]; in other studies T2D even tended to be protective [300, 301] although the latter result was not significant either on initial report or when reanalyzed in meta-analyses. Interestingly, those studies which resulted only in minimal increase in fracture risk involved mainly diet controlled [291, 302] or early onset T2D. Liefde et al. that showed that people with impaired glucose tolerance tended to have lower fracture risk (HR 0.8; 0.63–1.00), which increased in treated type 2 diabetics (HR 1.69; 1.16–2.46) despite an equally increased BMD [296]. Consistent with these data, the association between diabetes and hip fractures in the meta-analysis conducted by Janghorbani et al. became stronger when the cohorts with more than 10 years of follow-up were evaluated separately [283]. It is possible that early on in the natural history of the disease higher BMD may protect from fractures. On the other hand, when diabetes progresses, factors such as hyperglycemia, chronic complications, and the need for multidrug treatments may impair bone quality and/or increase the risk of falls and subsequently fractures. For example, retinopathy reduces vision, polyneuropathy alters gait, and cardiovascular complications lead to heart failure and cardiac arrhythmias, all factors promoting falls [303–305]. Diabetic nephropathy increased hip fracture risk 12-fold in patients with T1D [306] and the fracture risk in the women's health study was related to the presence of diabetic complications such as neuropathy and use of TZD (in postmenopausal women) and insulin in patients with T2D [307].

### 6.8. Relationship with Blood Glucose Control

Although pathophysiological evidence suggests that treating hyperglycemia may revert mechanisms associated with diabetic bone loss, intensive blood glucose control may increase the rate of hypoglycemic episodes and therefore falls. However, the relationship between glucose control and fractures is not clear with observational studies reporting mixed results. Most of the studies did not show any association between HbA1c or fasting glucose and fracture risk [286, 287, 290, 307]. However, in a recent Japanese study on men with T2D, vertebral fractures identified with spine films were associated with HbA1c >9% among those who were obese or overweight [308]. In a clinical trial on 50 patients with T2D with high HbA1c (mean 11.6%) followed up to 1 year, improved blood glucose control was followed by an increase in bone density at the neck, and the bone formation marker OCN was reduced after the treatment [309]. Recently, additional data were provided by the ACCORD BONE ancillary study [310]. The ACCORD trial compared tight blood glucose control targeting normal HbA1c levels (i.e., <6%) with standard strategy in a population with long-standing T2D and history of cardiovascular disease or high cardiovascular risk [311]. The ancillary BONE study found that intensive glycemia did not increase or decrease fracture or fall risk in comparison with the standard strategy [310]. Despite an increased rate of hypoglycemic events no increased fractures or fall were showed in this study [310]. Moreover, the author suggested that achieving lower HbA1c for several years might not be sufficient to reduce these risks in diabetes patients.

### 6.9. Antidiabetic Drugs and Risk of Fractures

Antidiabetic drugs may influence bone turnover but the effective role of these medications is not always clear. The main evidence available comes from post hoc analysis of blood-glucose lowering trials, where the effective role of these medications is often difficult to discern. The increased risk of fall associated with hypoglycaemic events and diabetic complications may indirectly confound the bone-specific effect associated with hypoglycaemic drugs. For example, preclinical and some clinical evidence in T1D suggest that insulin is protective against bone loss while most of the trials indicated an increased risk of fractures in insulin-treated T2D patients.

#### 6.9.1. Insulin

Most available studies report a higher incidence of bone fractures in insulin-treated patients, in comparison with non-insulin-treated T2D individuals. Monami et al. [312] published a case-controlled study in which the difference between control subjects and patients receiving long-term insulin treatment was analyzed. Within a cohort of 1,945 outpatients with diabetes with a follow-up of 4.1 ± 2.3 years, this study compared 83 cases of bone fractures and 249 controls matched for age, sex, duration of diabetes, BMI, levels of HbA1c, comorbidity, smoking, and alcohol abuse. Insulin-treated patients usually show a longer duration of diabetes and a higher prevalence of diabetes complications and it is possible that some studies, which did not provide adjustments for such confounders, could have overestimated the negative impact of insulin treatment. At the same time, treatment with insulin at the index date showed a significant association with bone fractures, maintained after adjusting for concomitant hypoglycemic medications. In fact the results showed that bone fractures in men were more frequent in insulin-treated patients (OR 3.20, 95%CI: 1.32–7.74), even if it was not confirmed in women (OR 1.41, 95%CI: 0.73–2.73). A study among older adults with diabetes showed that HbA1C <6% was related with greater risk of falls but only in those treated with insulin therapy [313]. These results are consistent with the hypothesis that insulin could increase the risk of fractures through falls caused by hypoglycemic episodes, without negative effects on bone metabolism. A recent analysis of the MrOS study evaluated the risk of nonvertebral fractures in 5,994 elderly men in relationship with diabetes status. In this study, diabetic men receiving insulin treatment had nearly double the risk of fractures compared with those without diabetes after adjustment for multiple covariates. In diabetic men who were not using insulin or in subjects with prediabetes, the fracture rate was not increased during an average 9-year follow-up [314].

#### 6.9.2. Thiazolidinediones

TZDs tackle insulin resistance via activation of PPAR*γ*. Clinical studies suggest that TZDs reduce BMD and increase fracture risk. Most of the evidence comes from studies on rosiglitazone. A post hoc analysis of the ADOPT (A Diabetes Outcome Progression Trial) [315], which was designed to compare the efficacy of rosiglitazone versus metformin and glyburide to maintain durable normal blood glucose levels in 1,840 women and 2,511 men with prediabetes [316], showed an increased risk of fractures associated with rosiglitazone [315]. This effect was evident in women but not in men with hazard ratios of 1.81 and 2.13 for rosiglitazone compared with metformin and glyburide, respectively. Fractures were seen predominantly in the lower and upper limbs, but vertebral fractures were not assessed in this study [315]. Bone resorption was increased in women and, although no increased fracture risk was showed in men, the bone formation marker P1NP was reduced in both genders [317].

Evidence from other clinical studies substantiated this finding showing a twofold increased risk of fractures in women taking TZD, but still showing no effect in men [318]. Considering that TZDs are PPAR*γ* agonists, a possible disruption in bone formation has been suggested. However, TZD are also known to lower RANKL activity and a recent study on ovariectomized rats indicates that bone impairment induced by rosiglitazone treatment is due to reduced bone strength coming from increased resorption mainly in sites rich in trabecular bone, which was reverted by treatment with alendronate [319]. Less evidence is available for pioglitazone. A recent study on 156 postmenopausal women with prediabetes showed that pioglitazone had no effect on BMD or bone turnover [320] with a similar result obtained by Grey et al. [321]. However, a meta-analysis of clinical studies has suggested an increased incidence of peripheral fractures in postmenopausal women with T2D taking pioglitazone [318].

#### 6.9.3. Metformin

Metformin is an insulin sensitizer and the most widely used oral hypoglycemic drug. It has been shown that AMPK activation by metformin may decrease expression of SERBP-1, a transcription factor involved in adipocyte differentiation and increased the activity f Runx2 enhancing osteoblastogenesis [322]. On the other hand, it has a negative effect on osteoclast differentiation by decreasing RANKL and increasing OPG levels [323]. Consistent with this finding, an analysis of the Rochester cohort suggested that biguanides may have a beneficial effect on bone fractures (HR 0.7; 95%CI: 0.6–0.95) [307]; however, analysis of the ADOPT did not show any beneficial effect [315] and in another trial metformin did not prevent rosiglitazone induced bone loss [324]. The MrOS study showed no effect of metformin on nonvertebral fracture risk in elderly men with diabetes [314].

#### 6.9.4. Sulphonylureas

Sulphonylureas work by stimulating insulin release by the *β*-cells. The post hoc analysis of the ADOPT study did not shown any effect of glyburide on fracture risk although treatment with this drug was associated with reduced bone formation marker P1NP [315]. In a recent analysis of the MrOS study, sulphonylurea use among elderly diabetic men was a risk factor for nonvertebral fractures (HR 1.66; 95%CI: 1.09–2.51) [314].

#### 6.9.5. Incretins

The incretin pathway is attenuated in T2D and the therapeutic target of drugs used in T2D such as GLP-1 receptor analogues and inhibitors of DPP-4, which extend the half-life or the native incretins. As stated above there is some* in vitro* and* in vivo* evidence that this peptides exert positive effect on bone. However, the clinical data are still scant. A recent meta-analysis, taking into consideration twenty-eight trials enrolling 11,880 patients on DPP-4 inhibitors, showed a trend to reduced risk of fractures (odds ratio [MH-OR] 0.60, 95%CI 0.37–0.99, *P* = 0.045) in these patients compared to those on placebo [325]. Indeed, another meta-analysis on seven trials showed that GLP-1 receptor analogues do not modify the risk of bone fractures in diabetes compared with the use of other antidiabetic drugs [326].

#### 6.9.6. Sodium-Glucose Cotransporters Inhibitors

Blockade of intestinal glucose uptake and renal glucose reabsorption via the sodium-glucose transporters (SGLT1, SGLT2) is a new approach to treat hyperglycemia in T2D [327]. Recently, the US Food and Drug Administration (FDA) approved two SGLT-2 inhibitors, canagliflozin and depagliflozin, as adjunctive therapy for T2D. These drugs inhibit selectively SGLT-2 in the kidney increasing urinary glucose excretion. Considering the mechanism of action, there is concern that renal tubular transportation of minerals and consequently bone health can be affected. In a 48-week trial assessing the efficacy of depagliflozin added on to pioglitazone in inadequately controlled T2D, Rosenstock et al. showed no clinically relevant changes in calcium, magnesium, phosphorous, or serum 25OHD levels [328]. A small increase in PTH and small mean changes in bone markers compared with placebo were shown. Two fractures occurred in the depagliflozin 5 mg group but all patients had received pioglitazone earlier [328]. In another study aimed to directly assess the effect of depagliflozin on bone turnover and BMD in patients inadequately controlled on metformin, Ljunggren et al. found no significant changes from baseline in P1NP, CTX, or BMD over 50 weeks of depagliflozin treatment [329]. Similar results were reported by Bolinder et al. [330]. In a pool analysis of eight clinical trials comprising 6,177 patients treated with canagliflozin, it was found that bone fracture incidence rates were 14.2, 18.7, and 17.6 per 1,000 patient years of exposure to comparator, canagliflozin 100 mg, and canagliflozin 300 mg, respectively. Patients treated with canagliflozin experienced more frequently extremity fractures than comparator [331]. On the other hand, a recent study on rats with a mixed SGLT1/2 inhibitor for 28 days resulted in marked changes in calcium and phosphorus homeostasis, suppression of PTH, 1.25(OH)_2_D, and bone turnover but had positive effects on bone mass and strength [332]. However, available data on bone health are not conclusive and FDA has required a postmarketing bone safety study that the companies must conduct as a condition for approvals of both canagliflozin and depagliflozin.

## 7. Summary and Practical Hints

**Figure 1:**
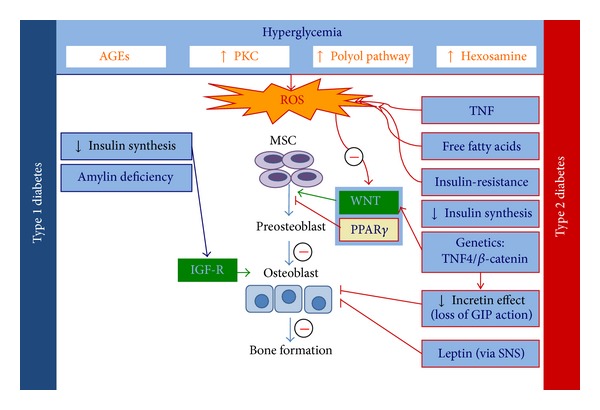
Diabetes-bone interaction. Several factors associated with diabetes may impair bone health. These factors may create, either directly or indirectly, a milieu that disrupts osteoblast differentiation and function, describing a low bone turnover phenotype. AGE: advanced glycation end-products; PKC: protein kinase C; ROS: reactive oxygen species; MSC: mesenchymal stem cells; TNF: tumor necrosis factor; GIP: gastric inhibitor peptide; IGF-R: insulin-like growth factor receptor; SNS: sympathetic nervous system.

Diabetes increases the risk of osteoporotic fractures through several mechanisms. Hyperglycemia impairs osteoblasts function, generates abnormal modifications of bone protein matrix, induces a state of chronic inflammation, and fuels diabetic complications that are associated with an increased risk of falls and fractures. Specific pathophysiological elements linked to the type of diabetes, such as insulin deficiency in T1D or loss of incretin effect in T2D, are also involved in impaired bone health. Finally, increased release of adipokines by fat tissue further manipulates bone homeostasis. Taken together, these factors create, either directly or indirectly, a milieu that promotes MSC fate toward adipogenesis over osteoblastogenesis, describing a low bone turnover phenotype (Figure 1).

Fracture prediction may be challenging especially in patient with T2D. In these patients, BMD values and FRAX score should be carefully interpreted. Subjects with T2D have increased fragility despite normal or high T-score; similarly, the fracture risk is higher when compared with nondiabetic people for a given FRAX score. DEXA limitations might be overcome by techniques that take into account bone size and geometry such as pQCT while additional factors need to be studied in order to generate better predictive algorithms.

Although hyperglycemia may fuel several mechanisms associated with bone loss, a tight blood glucose control may increase the rate of hypoglycemia and therefore falls. Unfortunately, there is no consistent evidence from clinical trials that tight blood glucose control either positively or negatively influences bone fractures. However, it is well known that an HbA1c <7% prevents chronic complications [333–336], especially in younger and uncomplicated patients, thus possibly reducing the associated risk of falls and fractures. Diabetes treatment may impact bone health and treatment decisions should be individualized. TZD should be avoided in postmenopausal women if possible and weight loss in patients with T2D should be accompanied by increased physical activity to prevent bone loss.

Impaired vitamin D status is more prevalent in diabetes than in people without diabetes. Intervention studies assessing the effectiveness of vitamin D and calcium supplementation on diabetes-related fractures are not available and no consensus has been reached on the optimal vitamin D serum level [337]. However, 25OHD serum levels >20 ng/mL are advisable [338] and there is evidence from broad population trials that higher concentrations reduce hip fractures by 23% [339] and fall risk by 19% [340].

Evidences on osteoporosis drugs in diabetes are also scant and limited to observational or post hoc analysis of bisphosphonates studies. Alendronate has been shown to be as effective in diabetes as in postmenopausal osteoporosis in increasing BMD [341] and preventing hip fractures [342] with no differences between T1D and T2D [342]. Considering the increasing evidence that suggests low bone formation in diabetes, osteoanabolic therapies such as PTH-based drugs are attractive [343] but this hypothesis has not been substantiated by clinical studies yet. Ad hoc trials with antiresorptive and anabolic drugs investigating fracture outcomes in diabetes are needed.

## Abbreviations

T2D: Type 2 diabetes
MSC: Mesenchymal stem cells
BMD: Bone mineral density
T1D: Type 1 diabetes
PPAR: Peroxisome proliferator-activated receptors
LRP: Low-density lipoprotein receptor-related protein
GSK: Glycogen synthase kinase
Dkk: Dickkoff
C/EBP: CCAAT/enhancer binding protein
RANKL: Receptor activator of nuclear factor-*κ*B ligand
NF-*κ*B: Nuclear factor-*κ*B
TNF: Tumor necrosis factor
OPG: Osteoprotegerin
BMI: Body mass index
pQCT: Peripheral quantitative computed tomography
IL-: Interleukin-
TNFR1: TNF receptor 1
ROS: Reactive oxygen species
OCN: Osteocalcin
11b-HSD: 11-*β*-hydroxysteroid dehydrogenase
DEXA: Dual-energy X-ray absorptiometry
PTH: Parathyroid hormone
1,25(OH)_2_D: 1,25(OH)_2_ vitamin D
25OHD: 25OH vitamin D
AGE: Advanced glycation end-products
CTX: Cross-linked C-telopeptide
IRS: Insulin receptor substrates
NOD mouse: Nonobese diabetic mouse
MRI: Magnetic resonance imaging
GIP: Glucose-dependent insulinotropic polypeptide
GLP-1: Glucagon-like peptide 1
DPP-4: Depeptidyl peptidase-4
ucOCN: Undercarboxylated osteocalcin
P1NP: Type 1 procollagen N-terminal peptides
COP: Circulating osteogenic precursor cell
TRAP: Tartrate-resistant acid phosphatase
BMC: Bone mineral content
FRAX: Fracture risk assessment tool
TBS: Trabecular bone score
RR: Relative risk
OR: Odds ratio
HR: Hazard ratio
HR-pQCT: High-resolution peripheral quantitative computed tomography
SGLT: Sodium-glucose transporters
FDA: Food and drug administration.
