# Corrigendum to “The Alliance of Mesenchymal Stem Cells, Bone, and Diabetes”

**DOI:** 10.1155/2017/5924671

**Published:** 2017-07-02

**Authors:** Nicola Napoli, Rocky Strollo, Angela Paladini, Silvia I. Briganti, Paolo Pozzilli, Sol Epstein

**Affiliations:** ^1^Division of Endocrinology and Diabetes, Università Campus Bio-Medico di Roma, Via Alvaro del Portillo 21, 00128 Rome, Italy; ^2^Division of Bone and Mineral Diseases, Washington University in St Louis, St Louis, MO, USA; ^3^Centre for Diabetes, The Blizard Building, Barts and The London School of Medicine, Queen Mary, University of London, London, UK; ^4^Division of Endocrinology, Mount Sinai School of Medicine, New York, USA

In the article titled “The Alliance of Mesenchymal Stem Cells, Bone, and Diabetes” [1], there was an error regarding the FRAX® tool, which should be clarified as follows:

The article notes: “Even the FRAX (fractures risk assessment tool), an algorithm adopted by the WHO to assess the risk of fractures, does not seem useful in T2D patients [186].” However, the World Health Organization (WHO) did not develop, test, or endorse the FRAX tool or its recommendations [2]. The metabolic bone disease unit at the University of Sheffield that developed FRAX was a WHO Collaborating Centre from 1991 to 2010, but treatment guidelines must undergo a formal process before they can be endorsed by the WHO.
